# CXCR3 chemokine receptor contributes to specific CD8^+^ T cell activation by pDC during infection with intracellular pathogens

**DOI:** 10.1371/journal.pntd.0008414

**Published:** 2020-06-23

**Authors:** Camila Pontes Ferreira, Leonardo de Moro Cariste, Isaú Henrique Noronha, Danielle Fernandes Durso, Joseli Lannes-Vieira, Karina Ramalho Bortoluci, Daniel Araki Ribeiro, Douglas Golenbock, Ricardo Tostes Gazzinelli, José Ronnie Carvalho de Vasconcelos

**Affiliations:** 1 Department of Microbiology, Immunology and Parasitology, Federal University of São Paulo, São Paulo, Brazil; 2 Department of Biosciences of the Federal University of São Paulo, Santos, Brazil; 3 Department of Medicine, University of Massachusetts Medical School, Worcester, Massachusetts, United States of America; 4 Laboratory of Biology of the Interactions, Oswaldo Cruz Institute, Fiocruz, Rio de Janeiro, Brazil; 5 Department of Biology Sciences, Federal University of São Paulo, São Paulo, Brazil; Ohio State University, UNITED STATES

## Abstract

Chemokine receptor type 3 (CXCR3) plays an important role in CD8^+^ T cells migration during intracellular infections, such as *Trypanosoma cruzi*. In addition to chemotaxis, CXCR3 receptor has been described as important to the interaction between antigen-presenting cells and effector cells. We hypothesized that CXCR3 is fundamental to *T*. *cruzi*-specific CD8^+^ T cell activation, migration and effector function. Anti-CXCR3 neutralizing antibody administration to acutely *T*. *cruzi*-infected mice decreased the number of specific CD8^+^ T cells in the spleen, and those cells had impaired in activation and cytokine production but unaltered proliferative response. In addition, anti-CXCR3-treated mice showed decreased frequency of CD8^+^ T cells in the heart and numbers of plasmacytoid dendritic cells in spleen and lymph node. As CD8^+^ T cells interacted with plasmacytoid dendritic cells during infection by *T*. *cruzi*, we suggest that anti-CXCR3 treatment lowers the quantity of plasmacytoid dendritic cells, which may contribute to impair the prime of CD8^+^ T cells. Understanding which molecules and mechanisms guide CD8^+^ T cell activation and migration might be a key to vaccine development against Chagas disease as those cells play an important role in *T*. *cruzi* infection control.

## Author summary

Inflammatory chemokine receptors such as CXCR3 play an important role in T lymphocytes migration into an infected tissue during Th1 response. Recently, the role of CXCR3 as a co-stimulatory molecule was demonstrated, and T lymphocytes from CXCR3 deficient mice had impaired effector function. CXCR3 receptor was highly expressed on specific CD8^+^ T cells after challenge with *T*. *cruzi*, and the hypothesis of that molecule is important for CD8^+^ T cells activation, migration and functionality was raised. We used the anti-CXCR3 neutralizing antibody approach and demonstrated that C57BL/6 treated mice died very quickly due to *T*. *cruzi* infection, and specific CD8^+^ T cells had decreased effector phenotyping, cytokine production, and cytotoxicity. In addition, anti-CXCR3 treatment decreased the number of dendritic plasmacytoid cells in the lymphoid tissues. The lower quantity of dendritic plasmacytoid cells in those tissues might contribute to the decrease in CD8^+^ T cells activation. Overall, CXCR3 molecule seems to be an important molecule to be explored during vaccine against Chagas disease strategies.

## Introduction

Chemokine receptors play an important role in T lymphocytes migration during homeostasis and inflammation. Inflammatory chemokines control the recruitment of effector leukocytes into infected tissues, and different types of these chemoattractant cytokines are preferentially expressed in innate and adaptive immune responses [1,2]. CXCR3 receptor, a G protein-coupled cell surface receptor (GPCR) with seven transmembrane α-helical domains, is expressed during Th1 adaptive response and it is an inflammatory chemokine inducible by CXCL9/MIG, CXCL10/IP-10 and CXCL11/I-TAC [3,4]. T-bet is a transcription factor that directly activates transcription of a set of genes which are important for Th1 cell function, including those encoding IFN-γ and the chemokine receptor CXCR3 [5].

CXCR3 receptor has been reported to be expressed in several immune cell types such as: T effector lymphocytes, CD4^+^ Foxp3^+^ T cells, natural killer (NK) and B cells [3,6]. We have demonstrated that CXCR3 is expressed in specific CD8^+^ T cells after *Trypanosoma cruzi* infection, which induced Th1 responses with high levels of IFN-γ cytokine [7]. In *T*. *cruzi* infection, it was demonstrated that patients with cardiomyopathy also had an increase in the expression of CXCR3 ligands [8,9].

The role of CXCR3 in T cell migration has been demonstrated during infection by *Toxoplasma gondii*: CXCR3^+^ CD4^+^ T migrated into infected tissues and controlled the infection [10]. In cardiac allografts rejection, T lymphocytes expressing CXCR3 are responsible for recruiting cytokine-producing T cells that cause inflammation, culminating with the allograft rejection [11]. During infection with parainfluenza, CXCR3 guides CD4^+^ T cells to the lungs [12]. Also, we demonstrated that CXCR3 is essential to specific CD8^+^ T cell migration into infected hearts and infection control during immunization with ASP-2-based anti-*T*. *cruzi* vaccine [13].

In addition to chemotaxis, CXCR3 signaling may influence the development of effector T cells because CD8^+^ T cells have reduced proliferative and cytotoxic ability in receptor- or ligand-deficient mice [14]. During infection with HSV-2, T cells from CXCR3-deficient mice exhibited impaired CD8^+^ T cell cytotoxicity and reduced expression of T-bet, IFN-γ, perforin and granzyme B [15].

Hickman and colleagues demonstrated that CXCR3-deficient CD8^+^ T cells had impaired cytotoxicity and suggested that CXCR3 plays a role in the contact between antigen-presenting cells and CD8^+^ T cells, allowing priming and activation of T cells [16]. In fact, CXCL10-expressing dendritic cells (DCs) actively interacted with T cells, indicating a role of CXCL10/CXCR3 in the interactions between the cells [17]. Recently, it was demonstrated that CXCL9 produced by allograft DCs promotes priming towards cytotoxic CD8^+^ T cells (CTL) and Th1 CD4^+^ IFN-γ producing T cells [18].

*T*. *cruzi*-specific CD8^+^ T cells, which are responsible for controlling parasite load via cytokine release and cytotoxic activity, express high levels of CXCR3 on the surface [19,20]. Therefore, we hypothesized that CXCR3 is pivotal for CD8^+^ T cell activation and effector function guiding those cells toward antigen-presenting cells, such as plasmacytoid dendritic cells (pDCs). We used the anti-CXCR3 neutralizing antibody approach to understand the role of CXCR3 in the CD8^+^ T cell function. For that propose, C57BL/6 mice were infected with *T*. *cruzi* and treated with anti-CXCR3 antibody every 48 hours, first, we evaluated the parasitemia and survival rate, also, we investigated the function, activation and migration of specific CD8^+^ T cells.

## Methods

### Ethics statement, mice, infection and treatments

We followed the recommendations in the Guide for the Care and Use of Laboratory Animals of the Brazilian National Council of Animal Experimentation to develop this study. The protocol (CEUA 7559051115) was approved by the Ethical Committee for Animal Experimentation at the Federal University of São Paulo. Eight-weeks-old female, C57BL/6, OT-I and Foxp3-GFP reporter mice were purchased from the Federal University of São Paulo (CEDEME). The REX3 lineage was provided by Dr. Ricardo Tostes Gazzinelli (University of Massachusetts). For infection of C57BL/6 (or background) mice, blood trypomastigotes forms of the Y strain of *T*. *cruzi* were maintained by weekly passages in A/Sn mice at the Xenodiagnosis Laboratory of Dante Pazzenese Cardiology Institute. For *in vivo* experiments using C57BL/6, each mouse was inoculated with 10^4^ trypomastigotes forms from blood (Y strain of *T*. *cruzi*) and OT-I mice were infected with 1x10^6^ Y-OVA transgenic parasites that were maintained in culture (LLCMK2 cells). Both parasites were diluted in 0.2 mL PBS and administered subcutaneously (s.c.) at the base of the tail. We used anti-mouse CXCR3 monoclonal antibodies (CXCR3-173, BioXcell) and Rat IgG2a isotype control antibody (clone 2A3, BioXcell). Both were administered via the intraperitoneal route on the same day of infection and every 48 hours until day 15 of infection. The quantity of antibodies administered was 250 μg of mAb/mouse, the same amount used by Uppaluri *et al* [21]. Parasitemia was determined by the examination of 5 μL of blood, and parasites were counted using a light microscope.

### Quantification of parasites burden and relative expression of CXCL10, CXCL9 and CD8 molecules

On day 15 after infection, hearts from the Isotype Control group and the anti-CXCR3 group were harvested, and DNA extraction was performed using phenol-chloroform-isoamyl alcohol (SIGMA). For Real Time PCR reaction, we used specific primers for a satellite DNA sequence of the parasite (*T*. *cruzi*) and TaqMan Universal Master Mix II with UNG, adapted from Piron and colleagues [22]. For quantification of chemokines, the RNA from mice’s heart was extracted using TRIzol and complementary DNA was prepared using Multiscript RT (Applied Biosystems). PCR was performed with SYBR Green Master Mix (Applied Biosystems) and the primer sequences used to measure chemokines and CD8 expression were: CXCL9, 5′-AATGCACGATGCTCCTGCA-3′ and 5′-AGGTCTTTGAGGGATTTGTAGTGG-3′; CXCL10, 5′-GCCGTCATTTTCTGCCTCA-3′ and 5′-CGTCCTTGCGAGAGGGATC-3′, CD8, 5′-GACGAAGCTGACTGTGGTTGA-3′ CD8, 5′-GCAGGCTGAGGGTGGTAAG-3′. The samples were normalized using GAPDH gene, 5′-GTGGTGAAGCAGGCATCTGA-3′ and 5′-GGGAGTCACTGTTGAAGTCGC-3′ primers.

### Image flow cytometry analyses

For the *in vivo* interaction experiment, C57BL/6 mice (CD45.1) were irradiated at 900 Rads. Each irradiated animal received 10×10^6^ bone marrow cells by i.v route, isolated from GREAT IFN-γ GFP reporter mice and REX3 CXCL10-BFP and CXCL9-RFP reporter mice (CD45.2). Twelve weeks after the transference, mice were infected with *T*. *cruzi* and, on day 12 after infection, spleens were harvested, then fixed with 1% of PFA, and labeled with DRAQ5 (BD, Pharmingen) and anti-CD8 (clone 53–67, BD). For experiments with REX3 mice, spleen cells were harvested on day 12 after infection, then fixed with 1% of PFA and labeled with: anti-CD8 (clone 53–67, BD), anti-CD11b (clone M1/70, ebioscience), anti-CD209a (clone MMD3, ebioscience), anti-CD317 (pDCA-1, Miltenyi Biotec), anti-CD11c (clone HL3, BD), and DRAQ5 (BD, Pharmingen). The samples were acquired from ImageStream Ammis, and we used the Ideas software to perform the analysis. The double cells were selected based on aspect ratio versus cell area [23].

### Immunological assays

The immunodominant peptide VNHRFTLV (pA8) from GenScript described earlier [24] was used for *ex vivo* stimulation splenocytes. For surface labeling, two million splenocyte cells were treated with lysis buffer and specific CD8^+^ T cells were labeled using H2K^b^-VNHRFTLV multimer (Immudex) for 10 minutes at RT. After that, the other surface antigens were labeled for 30 min at 4°C. The antibodies used were: anti-CD3 (clone 145-2C11, BD), anti-CD8 (clone 53–67, BD), anti-CD11a (clone 2D7, BD), anti-CD11c (clone HL3, BD), anti-CD44 (clone IM7, BD), anti-CD62L (clone MEL-14, BD), anti-CXCR3 (clone 173, BioLegend), anti-CD27 (clone LG3A10, BD), anti-CD4 (clone RM4-5, BD) anti-KLRG1 (clone 2F1, eBioscience), anti-CD49d (clone R1-2, BD), anti-CD69 (clone H1.2F3, BD), anti-CD43 (1B11, BioLegend), anti-CD95 (clone JO2, BD), anti-CD25 (clone LG3A10, BD), anti-CD127 (clone SB/199, BD), anti-CD122 (clone TM-β1, BD), anti-CD38 (clone 90, BD), anti-β7 (clone FIB27, BioLegend), anti-CD31 (clone MEC 13.3, BD), anti-CD272 (clone 8F4, eBioscience), anti-PD-1 (clone J43, eBioscience) and anti-CTLA-4 (clone UC10-4B9, eBioscience). For annexin V and 7-AAD assays, 2x10^6^ of splenocytes were stained according to the Annexin Kit protocol (BD Pharmingen). To measure cytokine profile, splenocytes were stimulated with peptide (pA8) for 12 hours, the supernatant was collected, and the cytokines were measured using the Cytometric Bead Array Mouse Th1/Th2/Th17 Cytokines Kit (BD).

For Intracellular Staining (ICS), 2x10^6^ of splenocytes were incubated during 12 hours with medium containing CD107a FITC antibody (clone 1D4B, BD), anti-CD28 (clone 37.51, BD), BD Golgi-Plug (1 μL/mL) and monensin (5 μg/mL) BD Golgi-Plug (1 μL/mL) and stimulated with 10 μM of peptide pA8. To detect IFN-γ, TNF or granzyme B, splenocytes were labeled with multimer and anti-CD8 PERCP antibody (clone 53–6.7, BD) on ice for 30 min. Following, the cells were fixed and permeabilized with BD perm/wash buffer. For intracellular staining, we used anti-IFN-γ (clone XMG1.2, BD), anti-TNF (clone MP6-XT22, BD), and anti-granzyme B (clone GB11, INVITROGEN). At least 700,000 cells were acquired on a BD FACS Canto II flow cytometer and then analyzed with FlowJo software.

For the ELISPOT assay, 1x10^6^ responder cells from the spleen were incubated with 3x10^5^ antigen-presenting cells in complete cell culture medium (1% NEAA, 1% L-glutamine, 1% vitamins and 1% pyruvate, 0,1% 2-ME, 10% FBS (HyClone), 20 U/ml mouse IL-2 recombinant (SIGMA) and, on a plate previously coated with 10 μg/ml of anti-IFN-γ capture antibody (clone R4-6A2, Pharmingen), those cells were incubated in the presence or absence of 10 μM of peptide pA8. After 24 hours, the plates were washed and incubated with 2 μg/ml of biotinylated anti-IFN-γ antibody (clone XMG1.2, Pharmingen). Subsequently, the plates were incubated with streptavidin-peroxidase (BD) and developed by adding peroxidase substrate (50mM Tris-HCl, pH 7.5, containing 1 mg/ml DAB and 1 μL/ml 30% hydrogen peroxide, both from SIGMA). The number of IFN-γ-producing cells was determined using a stereoscope.

### *In vivo* BrdU incorporation

BrdU (5-bromo-2'-deoxiuridine, SIGMA) administration was performed on the same day of infection; 2mg of BrdU were injected via i.p route every 48 hours until 15 days after infection. Then, 2x10^6^ splenocytes were stained according to the BrdU-FITC Kit protocol (BD Pharmingen) in order that the percentage of specific CD8^+^ T cells that incorporated BrdU could be analyze. At least 700,000 cells were acquired on a BD FACS Canto II flow cytometer and analyzed with FlowJo software.

### *In vivo* cytotoxicity assay

The protocol described by Silverio *et al* [25] was used. Splenocytes from naïve mice were divided into two populations labeled with CFSE (Molecular Probes) at a final concentration of 10 μM (CFSE^high^) or 1 μM (CFSE^low^). CFSE^high^ cells were coated with 2.5 μM of pA8 peptide for 40 minutes at 37°C. Subsequently, CFSE^high^ cells were mixed with equal numbers of CFSE^low^ cells before intravenous injection (2x10^7^ cells per mouse) into recipient mice. Spleen cells from the recipient mice were collected 18 hours after adoptive cell transfer and the events were acquired on a BD FACS Canto II flow cytometer and analyzed with FlowJo 8.7. The percentage of specific lysis was determined using the following formula:

$$\%\mathrm{lysis}=1-\frac{\left(\%{\mathrm{CFSE}}^{\mathrm{high}}\;\mathrm{infected}/\%{\mathrm{CFSE}}^{\mathrm{low}}\;\mathrm{infected}\right)}{(\%{\mathrm{CFSE}}^{\mathrm{high}}\;\mathrm{naive}/\%{\mathrm{CFSE}}^{\mathrm{low}}\;\mathrm{naive})\times 100}$$

### Histology and immunohistochemistry

Hearts from naïve, Isotype Control and anti-CXCR3 groups were fixed in 10% formalin, and then dehydrated, embedded in paraffin blocks, and sectioned in a microtome. Staining was performed using hematoxylin and eosin. The number of amastigote nests was quantified using a light microscope with a 40x objective lens. Overall, 50 fields/group were counted. For immunohistochemistry the mice’s hearts were frozen in Tissue-Tek O.C.T (Sakura Fineteck); the blocks were sectioned in cryostat (7μm thickness) and fixed in ice-cold acetone for 15 minutes. The samples were stained with 20 μg of the biotinylated anti-CD8 antibody (clone 53–6.7, RD systems) overnight, and labeled with streptavidin Alexa Fluor 488 (Thermo Fischer) at a concentration of 0.5 mg/mL for 1 hour at room temperature. The DAPI (SIGMA) dye was used for labeling the cell nucleus at a concentration of 5 mg/ml. The images were acquired on Confocal Leica TCS SP8 CARS microscope from the Institute of Pharmacology and Molecular Biology (INFAR). The images were obtained using a 63x objective lens and processed on ImageJ software.

### Statistical analysis

The data was presented as mean ± Standard Deviation (SD). Unidirectional Variance (ANOVA), the Tukey’s HSD posthoc and Student’s t-test (http://vassarstats.net/) were used to compare parasitemia, the number of IFN-γ-producing cells (ELISPOT), and absolute number of CD8^+^ T-cells. The survival rate was compared using the Log-rank test using GraphPad Prism 7. Molecule expression was compared using MFI (Mean Fluorescence Intensity), determined by the FlowJo software (version 10.5.3). Differences were considered statistically significant when *P*< 0.05.

## Results

### *T*. *cruzi* infection upregulates CXCR3 on T lymphocytes in lymphoid tissues and its ligands CXCL10 and CXCL9 in the heart tissue

**Fig 1:**
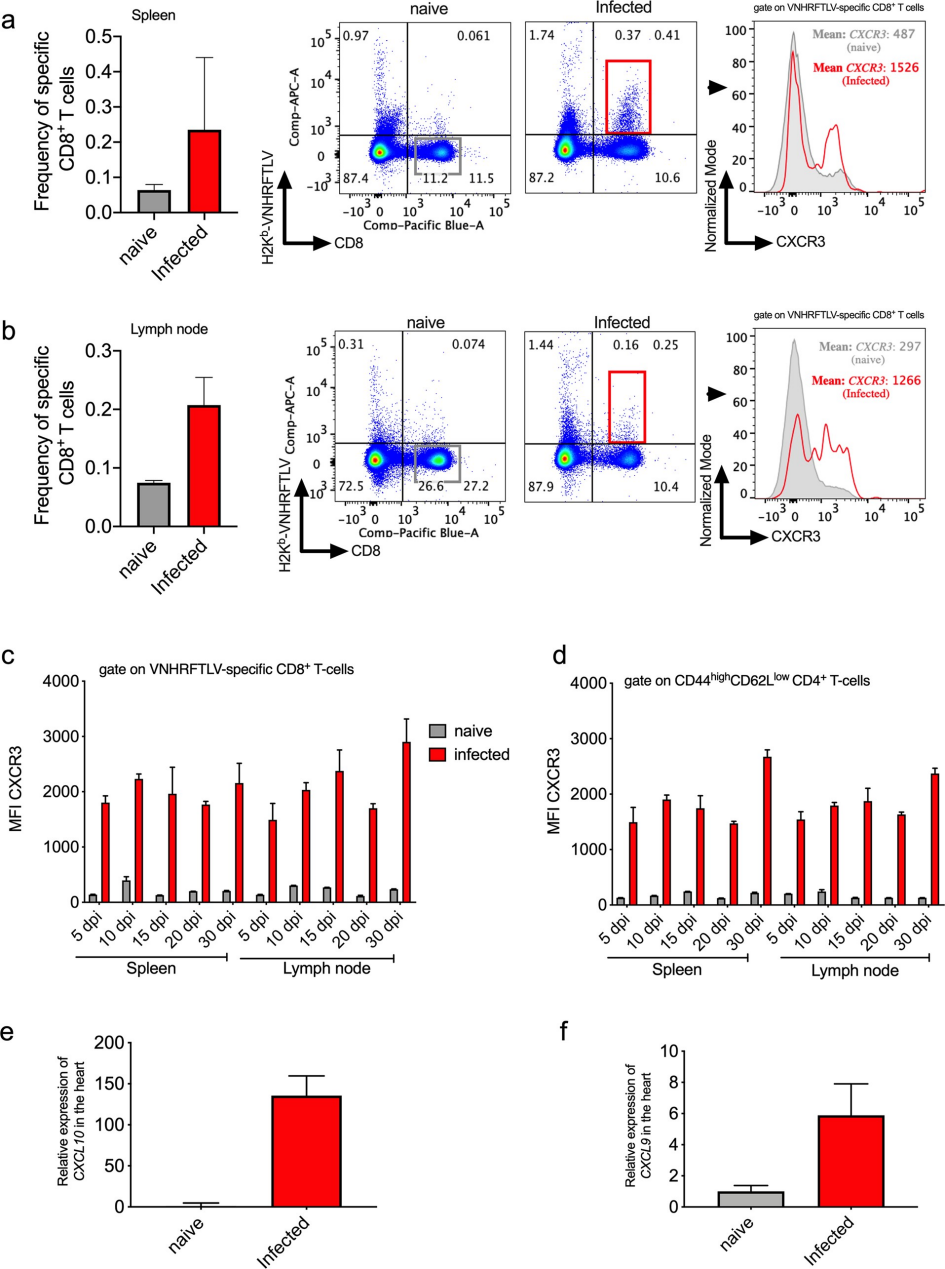
CXCR3 chemokine receptor is highly expressed on specific CD8^+^ and activated CD4^+^ T cell surfaces as well as the CXCR3 ligands in the heart of infected mice. C57BL/6 mice were infected with 1x10^4^ blood forms of the Y strain of *Trypanosoma cruzi*. a-The bar graph indicates the frequency of VNHRFTLV specific CD8^+^ T cells and the representative dot-plot graphs show the frequency of specific CD8^+^ T cells in the spleen. The histogram showed CXCR3 expression on specific CD8^+^ T in the spleen of naïve and infected mice. b-The bar graph indicates the frequency of VNHRFTLV specific CD8^+^ T cells and the representative dot-plot graphs show the frequency of specific CD8^+^ T cells in lymph node. The histogram showed CXCR3 expression on specific CD8^+^ T in the lymph node of naïve and infected mice 15 days post infection. The grey line represents the naïve animals while the red line represents the infected ones. c-The graphs represent MFI of CXCR3 in specific CD8^+^ and (d) activated CD4^+^ T cells. CXCR3 expression was evaluated on days 5, 10, 15, 20 and 30 post infection (dpi) in the spleen and lymph node. e-Relative expression of CXCL10 and (f) CXCL9 chemokines in naïve and infected mice hearts 15 days post infection. Data are mean ± SD and are representative of one independent experiment with n = 4.

Here, we checked whether specific CD8^+^ and activated CD4^+^ T cells generated by *T*. *cruzi* infection expressed CXCR3 receptor. For that propose, we labeled specific CD8^+^ T cells in the spleen and lymph nodes with multimer H2K^b^-restricted VNHRFTLV, an immunodominant peptide of ASP-2 *T*. *cruzi* surface amastigote protein [26,27]. To label CD4^+^ T cells, we first gated those cells as CD44^high^CD62L^low^ and then for CD4^+^ T cells. On the bar graphs, it can be observed the frequency of specific CD8^+^ T cells in the spleen and lymph nodes in infected mice on day 15 after infection (Fig 1A and 1B).

Likewise, VNHRFTLV specific CD8^+^ and activated CD4^+^ T cells were gated as showed in dot-plots graph and we observed that those cells expressed high levels of CXCR3 molecule on the surface of both spleen and lymph node, as shown by an increase in MFI numbers in comparison to naïve group (Fig 1A and 1B). Furthermore, CXCR3 expression was evaluated in the spleen and lymph nodes on days 5, 10, 15, 20, and 30 after infection. In specific CD8^+^ and activated CD4^+^ T cells, we observed that CXCR3 expression was high in both T cells and tissues on each day of analysis (Fig 1C and 1D).

In addition, CXCR3 ligand (CXCL10/IP-10 and CXCL9/MIG) expressions were evaluated in infected hearts by RT-PCR (Fig 1E and 1F). We noticed an increase in CXCL10 and CXCL9 expressions during infection compared to *naïve* hearts. Further, the relative expression of CXCL10 was higher than CXCL9 in infected mice. Collectively, these data showed that CXCR3 and their CXCL10 and CXCL9 ligands were highly expressed during acute *T*. *cruzi* infection.

### Anti-CXCR3 treated mice increased parasitemia and susceptibility to *T*. *cruzi* infection in C57BL/6 mice

**Fig 2:**
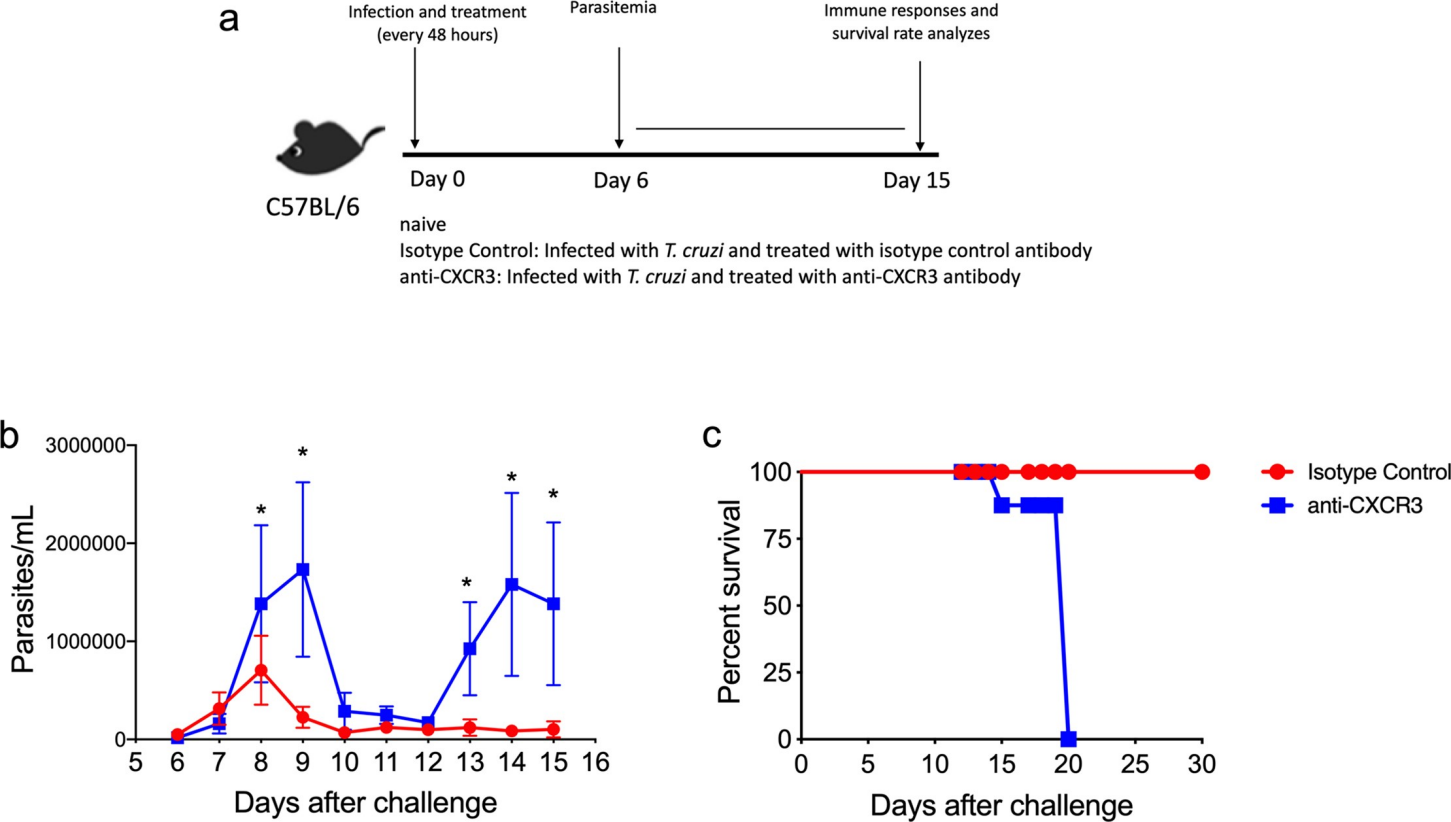
CXCR3 receptor is important to parasitemia and survival of C57BL/6 infected with *Trypanosoma cruzi*. a-Experimental scheme showing the model used in this study. Both Isotype control and anti-CXCR3 antibody treated groups were infected with 1x10^4^ blood trypomastigotes of the Y strain of *T*. *cruzi*. On the same day of infection, anti-CXCR3 group was treated with 250 μg of anti-CXCR3 antibody while the isotype control group was treated with the same concentration of anti-IgG of Rat antibody. The treatment was performed every 48 hours until 15 days after infection by intraperitoneal route. b-Graph of parasitemia showed in linear scale of Isotype Control and anti-CXCR3 groups. c-The Kaplan–Meier curves for survival of Isotype control and anti-CXCR3 groups. The statistical differences between the groups were compared using the log-rank test (p = 0.0091). Data are mean ± SD and are representative of 3 independent experiments with n = 4. *values are significantly different between isotype Control and anti-CXCR3 groups in indicated days (*p>0.001). The statistical analysis was performed using the Student’s t-test.

Here, we assessed whether CXCR3 receptor is important to control *T*. *cruzi* infection in C57BL/6 mice. For that intend, C57BL/6 mice were infected and on the same day treated with 250 μg of anti-CXCR3 or IgG2a isotype control antibodies. The treatment was repeated every 48 hours and for two weeks. On day 15 after infection, we performed immune responses and survival rate analyses as shown in the experimental scheme (Fig 2A). During the infection with the Y strain of *T*. *cruzi*, the peak of parasite levels in the blood of C57BL/6 mouse usually occurs on day 9 after infection. After that, this mouse strain controls the acute phase and the number of parasites drastically decreased after the peak as we described previously [28]. As we can see in the Fig 2B, mice treated with isotype control antibody displayed low levels of parasitemia and did not change the already described parasitemia profile after infection with the Y strain of *T*. *cruzi*. Interestingly, on day 9 after infection (the peak), the anti-CXCR3 treated group had higher parasitemia compared to the Isotype control group (Fig 2B). After the peak, parasitemia in isotype control and anti-CXCR3 treated groups decreased, and on day 13, the parasitemia levels increased again only in anti-CXCR3 treated group (Fig 2B). The parasitemia level in the anti-CXCR3 group remained high until day 15 after infection in contrast with the Isotype control group (Fig 2B). These results indicate that anti-CXCR3 treated mice could not control the parasite multiplication. In addition, all anti-CXCR3 treated mice which presented high parasitemia became susceptible and died very quickly due to infection when compared to controls, which had a 100% survival rate (Fig 2C). Together, those results indicate that CXCR3 receptor is important to control *T*. *cruzi* infection in C57BL/6 mice.

### The number of H2K^b^-restricted VNHRFTLV-specific CD8^+^ T cells decreased as well as polyfunctionality and cytotoxicity after anti-CXCR3 treatment

**Fig 3:**
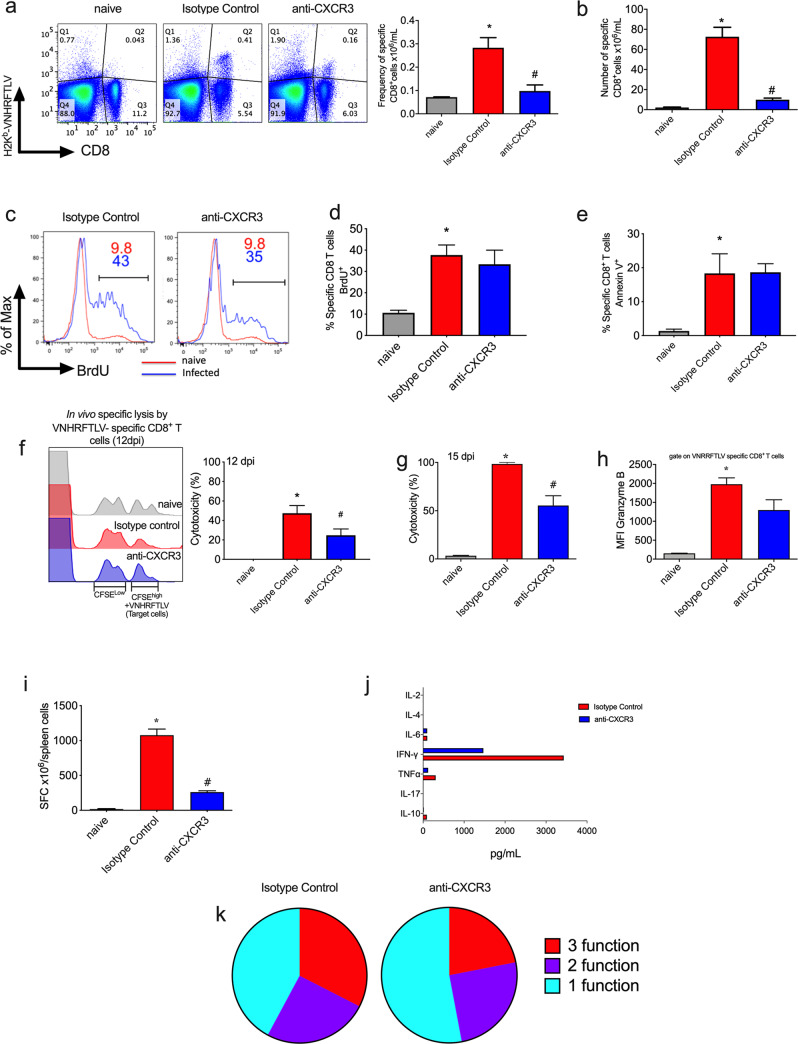
Anti-CXCR3 group decreased number of specific CD8^+^ T cells as well as polyfunctionality after infection with *T*. *cruzi*. a-Representative dot-plot graphs of each group show the frequency of specific CD8^+^ T cells (in Q2 quadrant) in the spleen and the bar graph represents the mean of specific CD8^+^ T cells frequency on day 15 after infection. Those cells were labeled with H2K^b^-VNHRFTLV multimer. b-The bar graph with absolute numbers of specific CD8^+^ T cells in the spleen on day 15 after infection. c-Histograms represent the gate on specific CD8^+^ T cells positive for BrdU; the red line represents one mouse from the naïve group while the blue one refers to the *T*. *cruzi* infected mice (Isotype control and anti-CXCR3 groups, respectively). d-Percentage of specific CD8^+^ T cells positive for BrdU (thymidine analogues) in the spleen. The *in vivo* proliferation assay was performed by BrdU administration (5mg/mouse). e-Percentage of specific CD8^+^ T cells in the spleen positive for Annexin V. f-The histogram represents the gate for CFSE^Low^ and CFSE^high^ (target cells) population in the spleen from naïve, Isotype control and anti-CXCR3 groups on day 12 post infection (12 dpi) and the bar graph represents the cytotoxicity percentage of specific CD8^+^ T cells from naïve, Isotype control and anti-CXCR3 groups. g-The percentage of cytotoxicity of specific CD8^+^ T cells from the spleen on day 15 post infection. h-The Mean Intensity of Fluorescence (MFI) of granzyme B on specific CD8^+^ T cells surface. i-Number of IFN-γ producing cells quantified by ELISPOT assay. j-Quantity of cytokines (pg/mL) from splenocytes supernatants. CBA mouse Th1/Th2/Th17 cytokine kit was used. k-Pie graphs show the percentage of specific CD8^+^ T cells that performed each combination as shown in legends (3, 2 or 1 function). The polyfunctionality of specific CD8^+^ T cells was performed after ICS staining and Boolean analyses. Data are mean ± SD and represent 3 independent experiments with n = 3. *P<0.05 comparing noninfected controls (naïve), Isotype control and anti-CXCR3 groups. # P<0.01 comparing Isotype control and anti-CXCR3 groups.

Specific CD8^+^ T cells play an important role in controlling *T*. *cruzi* infection by cytokine production and cytotoxicity against infected cells [20]. Initially, we evaluated the number of specific CD8^+^ T cells in spleen after anti-CXCR3 treatment on day 15 after infection. We observed a percentage 0.2% of specific CD8^+^ T cells in isotype control treated mice whereas the anti-CXCR3 treated mice group showed 0.09% of specific CD8^+^ T cells (Fig 3A). Consequently, the absolute number of specific CD8^+^ T cells in the anti-CXCR3 treated group drastically decreased compared to the isotype control group (Fig 3B). Additionally, we analyzed the number of total CD4^+^ T cells and we could observe that the treatment with anti-CXCR3 antibody did not alter the absolute number of CD4^+^ T cells in the spleen when compared to the isotype control group (S1A Fig). Moreover, we evaluated the number of CD4^+^ T cells Foxp3^+^ in the spleen using Foxp3-GFP reporter mice and, despite the decrease in frequency of CD4^+^Foxp3^+^ cells in the isotype control (0.19%) and anti-CXCR3 (0.23%) groups when compared to naïve cells (2.23%), we did not observe any statistical differences in the absolute number of CD4^+^Foxp3^+^ cells among naïve, isotype control and anti-CXCR3 groups (S1B and S1C Fig). The CD4^+^ Foxp3^+^ cells from infected groups expressed high levels of CD25 molecule on the surface (S1 Fig). Furthermore, CXCR3 receptor was highly expressed on the surface of CD4^+^ Foxp3^+^ cells during infection when compared to naïve cells (S1E Fig).

Next, we examined whether specific CD8^+^ T cells could proliferate after the anti-CXCR3 antibody treatment. To perform that, we administered BrdU molecule (thymidine analogues) on the same day of infection and quantified the incorporation of that molecule by flow cytometry. We observed that, after infection with *T*. *cruzi*, the percentage of specific CD8^+^ T cell BrdU^+^ increased when compared to naïve CD8^+^ T cells (Fig 3C). Also, the percentages of specific CD8^+^ T cells that were BrdU positive from Isotype control and anti-CXCR3 groups were similar (Fig 3D), indicating that the anti-CXCR3 treatment did not alter the proliferation of specific CD8^+^ T cells in the spleen. Furthermore, we measured the pro-apoptotic profile of specific CD8^+^ T cells after anti-CXCR3 treatment by using annexin V labeling. There was an increase of specific CD8^+^ T cells positive for annexin V after infection when compared to CD8^+^ T cells from naïve group. However, we did not observe any differences between the percentage of specific CD8^+^ T cells positive for annexin V between isotype control and anti-CXCR3 groups (Fig 3E).

Another important function triggered by CD8^+^ T cells is the directed cytotoxicity against infected cells. It is known that during *T*. *cruzi* infection there is an induction of cytotoxic CD8^+^ T cells. Silverio and colleagues demonstrated two populations of anti-*T*. *cruzi* CD8^+^ T cells segregated into a group showing the capacity for producing interferon-gamma (IFN-γ) and another one that performed specific cytotoxicity [25]. Thus, we performed the *in vivo* cytotoxicity of specific CD8^+^ T cells on day 12 after infection and observed that those cells from the Isotype control mice had almost 50% of cytotoxicity, while the percentage in the anti-CXCR3 group was 27% (Fig 3F). Surprisingly, on day 15 after infection, the cytotoxicity of infected specific CD8^+^ T cells was 100% in the isotype control group and in the anti-CXCR3 group the cytotoxicity decreased almost 50% percent when compared to the isotype control group (Fig 3G). We also examined granzyme B production, which is one of the important cytotoxic granules produced and released by CD8^+^ T cells. After infection, specific CD8^+^ T cells increase granzyme B expression when compared to CD8^+^ T cells from naïve group. In the anti-CXCR3 group we detected a decrease in MFI of granzyme B compared to isotype control group, but that decrease was not statistically different (Fig 3H).

In addition, cytokine production by specific CD8^+^ T cells was analyzed by ELISPOT, CBA and ICS assays. Using ELISPOT assay, harvested splenocytes from naïve, isotype control and anti-CXCR3 groups were stimulated with pA8 peptide and the number of CD8^+^ IFN-γ producing cells was counted. We observed that the anti-CXCR3 group had a decrease in the number of IFN-γ CD8^+^-producing cells in the spleen when compared to the isotype control group (Fig 3I). Additionally, using CBA assay, we measured Th1/Th2/Th17 cytokine profile from splenocytes supernatant and observed a high quantity of cytokines related to Th1 response in the infected groups, such as IFN-γ and TNF, however, we noticed that in the anti-CXCR3 group it was a trend in IFN-γ decrease (Fig 3J).

We also checked the polyfunctionality of specific CD8^+^ T cells measured by ICS. We quantified the percentage of specific CD8^+^ T that were degranulating (CD107a) and/or producing TNF and/or IFN-γ. Those cells were subdivided into groups of cells that were triggered 3, 2 or 1 function. We observed that the percentage of specific CD8^+^ T triple positive cells in the Isotype Control group was 3.83% while that percentage dropped to 0.47% in the anti-CXCR3 group, indicating that the anti-CXCR3 treatment diminished the polyfunctionality of specific CD8^+^ T cells (Fig 3K). In order to confirm the decrease in polyfunctionality of specific CD8^+^ T cells during the anti-CXCR3 treatment, we infected OT-I mice with a transgenic parasite, Y strain which expresses OVA protein. On the same day of infection, we treated the mice with anti-CXCR3 antibody. Subsequently, splenocytes were stimulated with SIINFEKL peptide for 12 hours and after that using ICS technique, IFN-γ, TNF and CD107a were labeled. Once again we observed that specific CD8^+^ T cells from mice treated with anti-CXCR3 antibody (OT-I+Y-OVA+anti-CXCR3 group) had a decrease in the percentage of specific CD8^+^ T cells triple positive when compared to mice treated with isotype control (OT-I+Y-OVA+isotype control group) (S2A and S2B Fig). Altogether those results indicate that CXCR3 receptor is important to cytotoxicity and cytokine production by specific CD8^+^ T cells.

### CXCR3 molecule contributes to anti-*T*. *cruzi* CD8^+^ T cells activation

**Fig 4:**
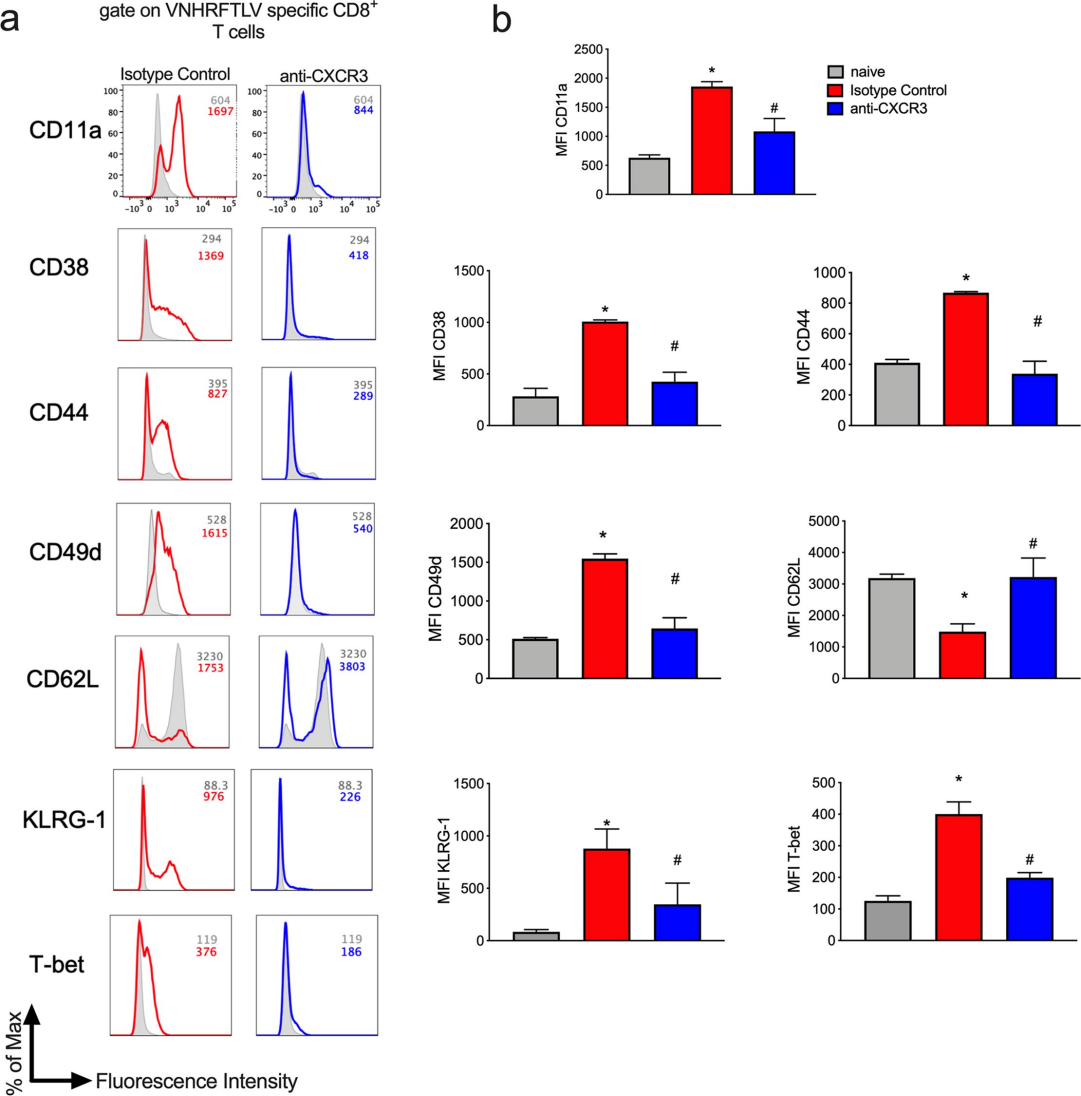
CXCR3 contributes to specific CD8^+^ T cells activation. The immunophenotyping of specific CD8^+^ T cells was performed in the spleen. We evaluated the expression of markers associated with activation, homing and memory. a-The histogram graphs represent each molecule analyzed from naïve (grey line), Isotype Control (red line) and anti-CXCR3 (blue line) groups. b-The bar graphs show MFI of each molecule analyzed in specific CD8^+^ T cells from naïve, Isotype Control and anti-CXCR3 groups. Data are mean ± SD and represent 2 independent experiments with n = 3. *P<0.05 comparing noninfected controls (naïve), Isotype Control and anti-CXCR3 groups. #P < .01 comparing Isotype control and anti-CXCR3 groups. The statistical analysis was performed using One-way ANOVA, followed by Tukey post-hoc test.

After activation, effector anti-*T*. *cruzi* CD8^+^ T cells upregulated the expression of CD11a, CD44, and KLRG-1 molecules and downregulated the expression of CD62L compared to naïve cells [29]. Since CXCR3 molecule is important to T cells differentiation [30], we examined the expression of activation, homing, or memory markers on specific CD8^+^ T cells after *T*. *cruzi* infection and anti-CXCR3 treatment. Those markers and expression levels are critical for the activation of specific CD8^+^ T cells, their migration into infection sites, and elimination of parasites. We compared the expression of molecules using the MFI (Mean of Fluorescence Intensity) and found that several molecules analyzed, such as CD11c, CD25, CD27, CD31, and CD122, did not change the expression levels both in the Isotype control group and anti-CXCR3 group (S3 Fig). However, some molecules were expressed differently by specific CD8^+^ T cells from those groups. Specific CD8^+^ T cells from the Isotype Control group highly expressed CD11a (LFA-1), CD38, CD44, CD49d, KLRG-1 and the transcription factor T-bet, and downregulated CD62L molecule when compared to naïve CD8^+^ T cells. On the other hand, specific CD8^+^ T cells from the anti-CXCR3 group downregulated CD11a (LFA-1), CD38, CD44, CD49d, KLRG-1 and T-bet, and upregulated CD62L molecule when compared to the Isotype Control group (Fig 4A and 4B).

Altogether, those results suggest that CXCR3 receptor may have a role in CD8^+^ T cell activation after *T*. *cruzi* infection.

### CXCR3 guides the migration of specific CD8^+^ T cells into infected hearts

**Fig 5:**
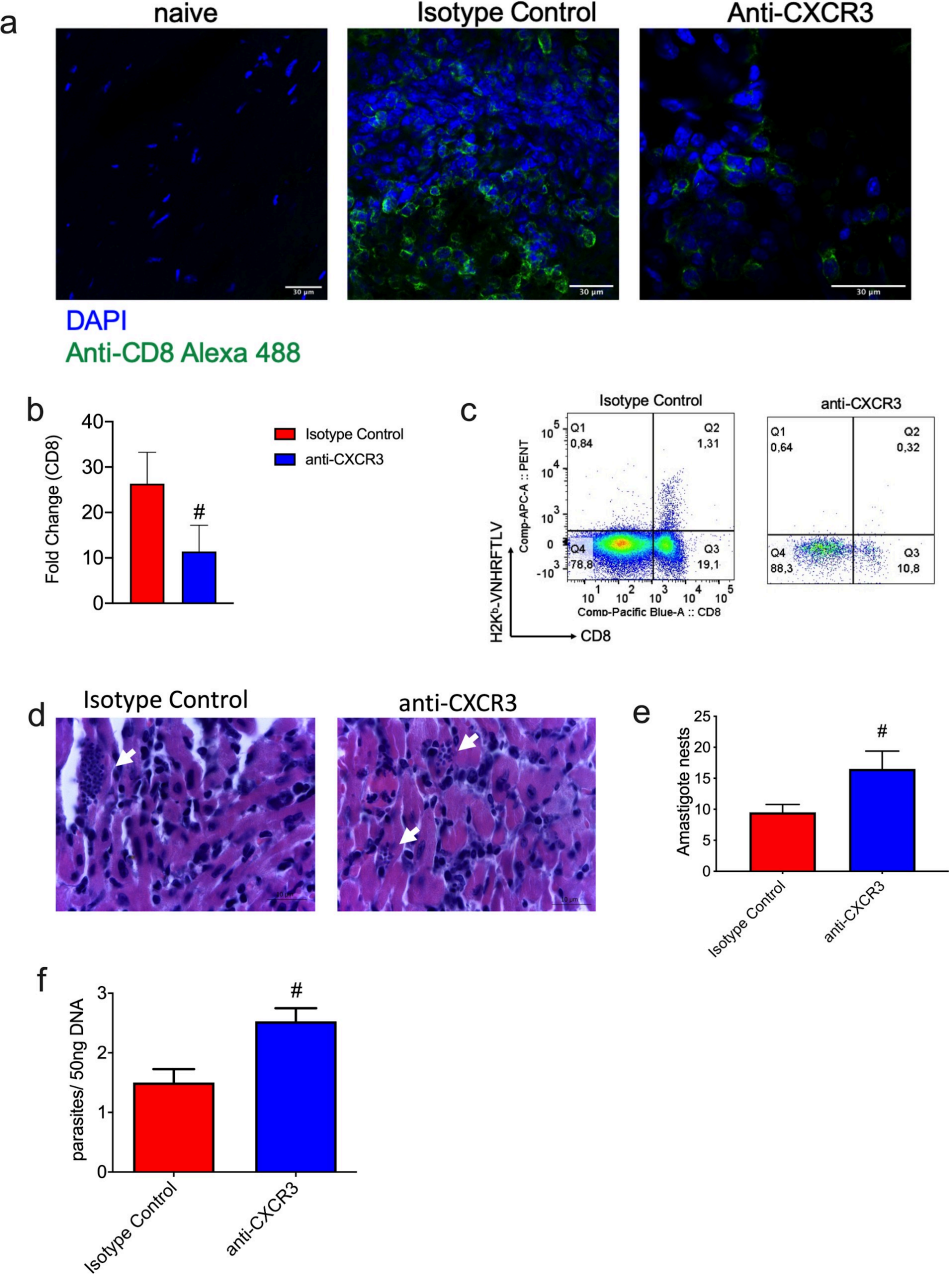
CXCR3 guides CD8^+^ T cells migration into *T*. *cruzi* infected heart. a-Immunohistochemistry staining of hearts from naïve, Isotype Control and anti-CXCR3 groups; CD8^+^ T cells were labeled in green. b-The relative expression of CD8^+^ T cells in the heart of Isotype Control and anti-CXCR3 groups. c-The dot-plot graphs represent the frequency of specific CD8^+^ T cells (pool of 3 mice/group) in the heart of Isotype Control and anti-CXCR3 groups. d-Histological section from infected hearts; the white arrows indicate the amastigote nests. e-Number of amastigote nests from Isotype Control and anti-CXCR3 groups counted in 50 fields. f-The parasite burden quantification by qPCR from infected hearts. The qPCR was performed from 50 ng of the heart’s DNA. Data are mean ± SD and represent 2 independent experiments. #P < .01 comparing Isotype control and anti-CXCR3 groups. The statistical analysis was performed using Student’s t-test.

Previously, our group described that CXCR3 is critical for migration of specific CD8^+^ T cells into the heart during vaccination with ASP-2 and infection with *T*. *cruzi* [13]. Firstly, we analyzed the presence of CD8^+^ T cells in the heart using confocal microscopy, and differently from naïve hearts, we observed CD8^+^ T cells (green) in the heart of Isotype Control and anti-CXCR3 groups (Fig 5A). However, in anti-CXCR3 group, we found fewer cells. In order to quantify the number of CD8^+^ T cells in the heart of both infected groups, we measured the relative expression of CD8 molecule by RT-PCR and observed a statistical decrease in CD8 expression in the heart of anti-CXCR3 treated mice when compared to the Isotype Control group (Fig 5B).

To confirm those data, we quantified the number of specific CD8^+^ T cells in the heart by flow cytometry; the specific CD8^+^ T cells were labeled with VNHRFTLV multimer. In the heart of the Isotype Control group, we observed 1.31% of specific CD8^+^ T cells while the percentage of specific CD8^+^ T cells in the anti-CXCR3 group was 0,32% (Fig 5C).

We investigated the tissue burden in the heart of infected mice and as expected, due the lower number of CD8^+^ T cells in the heart of anti-CXCR3, we observed an increase of amastigote nests in the anti-CXCR3 group when compared to the Isotype Control group (Fig 5D and 5E). In addition, the number of parasites (on day 15 after infection) in the heart was quantified by qPCR. We observed higher parasite numbers in the anti-CXCR3 group when compared to the Isotype Control group (Fig 5F).

Altogether, these results indicate that CXCR3 is important to specific CD8^+^ T cells migration into the heart of *T*. *cruzi* infected mice and to control the infection.

### pDCs produced CXCL10 chemokine and interacted with CD8^+^ T cells after infection with *T*. *cruzi*

**Fig 6:**
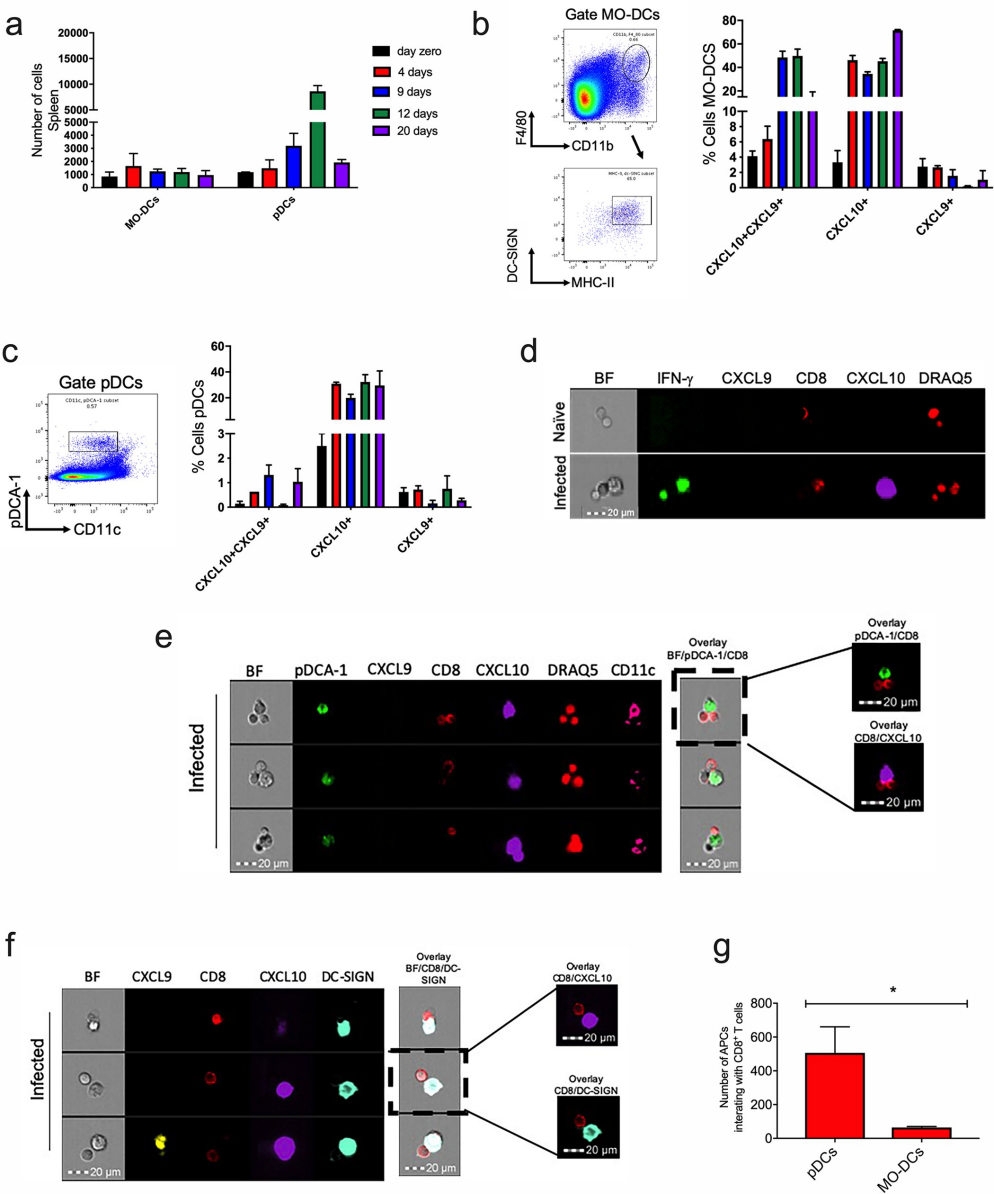
Plasmacytoid dendritic cells (pDCs) produced CXCL10 chemokine and interacted *in vivo* with CD8^+^ T cells during *T*. *cruzi* infection. To analyze which antigen-presenting cells (APCs) produced CXCR3 ligands, a REX3 linage mouse (CXCL10-BFP and CXCL9-RFP reporter) was infected on days 4, 9, 12, and 20 and we analyzed the number of MO-DCs, pDCs and chemokine production. a-The graphs represent the number of MO-DCs and pDCs on days 0, 4, 9, 12, and 20 after infection. b-The MO-DCs were gated as F4/80^+^CD11b^+^ and DC-SIGN^+^MHC-II^+^; the graph represents the percentage of MO-DCs-producing chemokines—CXCL9 and/or CXCL10. c-The pDCs were gated as CD11c^low^ and pDCA-1^+^ and the graph represents the percentage of pDCs producing chemokines—CXCL9 and/or CXCL10. d-Image Flow Cytometry of chimera mice that received IFN-γ GFP, CXCL9-RFP and CXCL10-BFP cells; the double cells were gated using DRAQ5 labeling. e-Image Flow Cytometry of REX3 infected mice shows the markers used as well as the interaction between CXCL10 pDC-producing cells and CD8^+^ T cells. f-Image Flow Cytometry of REX3 infected mice shows the markers used as well as the interaction between CXCL10 MO-DC-producing cells and CD8^+^ T cells. g-The number of pDCs and MO-DCs interacting with CD8^+^ T cells after *T*. *cruzi* infection. Data are mean ± SD and represent one experiment with n = 3. *indicates statistical differences between the number of pDCs and MO-DCs interacting with CD8^+^ T cells (*P = 0.001515, statistical analysis was performed using Student’s t-test).

CXCR3 ligands are produced by antigen-presenting cells (APCs) [30]. During cerebral malaria, the monocyte-derived dendritic cells (MO-DCs) produce CXCL10 and CXCL9 [31], but it is still unclear which APCs are responsible for producing CXCR3 ligands during *T*. *cruzi* infection. Previously, our group demonstrated that dendritic plasmacytoid cells (pDCs) have a role in *T*. *cruzi* antigen presentation [32]. Here, we investigated whether pDCs and MO-DCs produced CXCR3 ligands. In order to analyze that, REX3 reporter mice (CXCL10-BFP and CXCL9-RFP) were infected and, on days 4, 9, 12, and 20 after infection, we quantified the number of those cells by flow cytometry. We observed that the number of MO-DCs increased on day 4 after infection only. However, in the pDCs population, we observed a higher number on day 12 after infection (Fig 6A). In addition, both MO-DCs and pDCs produced CXCL10 chemokine on days 4, 9, 12, and 20 after infection (Fig 6B and 6C), but only MO-DCs produced CXCL9 and CXCL10 at the same time (Fig 6C). To investigate which CXCL10^+^ antigen-producing cells might interact *in vivo* with IFN-γ−producing cells after *T*. *cruzi* infection, we generated bone marrow chimeras. Irradiated WT mice were reconstituted with bone marrow from Great IFN-γ GFP reporter mice and REX3 CXCL10-BFP and CXCL9-RFP reporter mice. After 12 weeks, those mice were infected with *T*. *cruzi* and, on day 12 after infection, the spleens were harvested. The interaction was evaluated using Image Flow Cytometry (ImageStream). To quantify the interaction among those cells, we gated the double cells labeled with DRAQ5 (nucleus marker) and then we gated for IFN-γ^+^/CXCL10^+^ or CD8^+^/CXCL10^+^ positive cells. As expected, the cells from naïve chimera mice did not produce IFN-γ and CXCL9/CXCL10; however, in the infected group, we could observe an interaction between CXCL10-producing cells and IFN-γ-producing cells (Fig 6D).

Next, we examined the interaction between MO-DCs and pDCs with CD8^+^ T cells after infection. The REX3 reporter mice were infected and, on day 12 after infection, the numbers of MO-DC^+^/CD8^+^ and pDC^+^/CD8^+^ double cells were calculated. The pDC population was gated as pDCA-1^+^ and CD11c^+^, and MO-DC population was gated first to CD11b^+^F4/80^+^ and then to DC-SIGN^+^ and MHC-II^+^. We observed that both pDCs and MO-DCs produced CXCL10 chemokine (in purple) and interacted with CD8^+^ T cells (in red) during the infection with *T*. *cruzi* (Fig 6E and 6F); however, the number of pDCs interacting with CD8^+^ T cells was higher than MO-DCs (Fig 6G).

**Fig 7:**
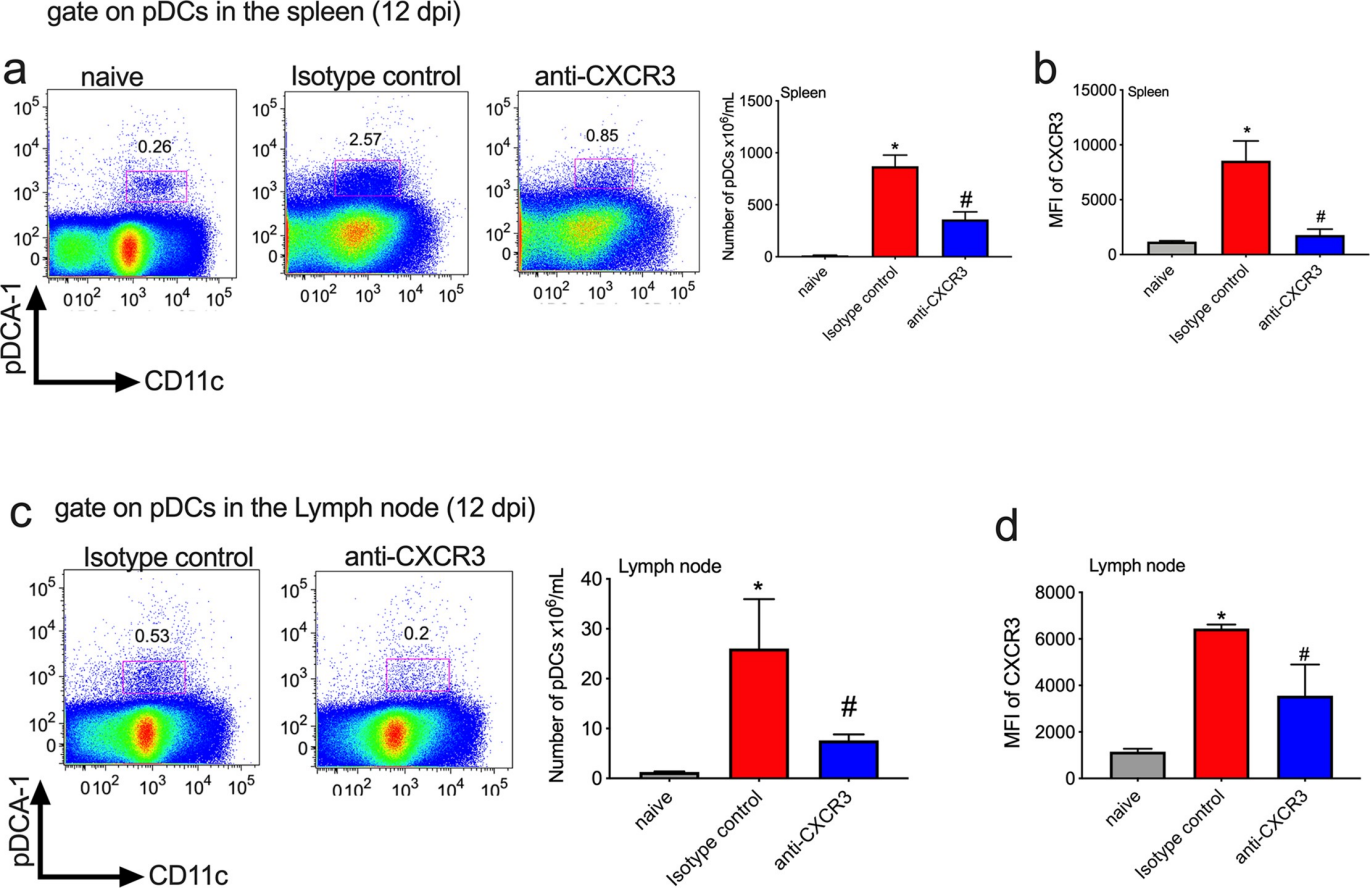
The number of plasmacytoid dendritic cells (pDCs) decreased after anti-CXCR3 treatment and *T*. *cruzi* infection. The number of pDCs after anti-CXCR3 treatment was quantified on day 12 after infection in the spleen and lymph node. a-The dot-plots represent the gate strategy used to measure the frequency of pDCs in the spleen and the bar graph represents the number of pDCs in the spleen of naïve, Isotype control and anti-CXCR3 groups. b-The MFI of CXCR3 receptor on pDCs surface in the spleen of naïve, Isotype control and anti-CXCR3 groups. c-The dot-plots represent the gate strategy used to measure the frequency of pDCs in the lymph node and the bar graph represents the number of pDCs in the lymph node. d-The MFI of CXCR3 receptor on pDCs surface on the lymph node of naïve, Isotype control and anti-CXCR3 groups. Data are mean ± SD and represent one experiment with n = 3. *P<0.05 comparing noninfected controls (naïve), Isotype control and anti-CXCR3 groups. #P < .01 comparing Isotype control and anti-CXCR3 groups.

Additionally, we analyzed the number of pDCs after the anti-CXCR3 treatment. We analyzed those cells on day 12 after infection because we observed a high number of pDCs in the spleen on that day. Interestingly, we observed that the number of pDCs in the spleen and lymph node decreased after infection and treatment with anti-CXCR3 antibody when we compared to the isotype control group (Fig 7A and 7B); also, pDCs expressed CXCR3 receptor on the surface after infection (Fig 7C and 7D).

## Discussion

Chemokine receptors guide T lymphocytes during homeostasis and infection. Also, may play a role in the activation and functionality of T lymphocytes [33,34]. CXCR3 chemokine receptor is highly expressed on T lymphocytes surface during Th1 responses against intracellular infection, such as with *Trypanosoma cruzi*. Previously, our group demonstrated that CXCR3 chemokine receptor was important to protect A/Sn vaccinated mice against death after *T*. *cruzi* infection [13]. Here, we aimed to examine whether CXCR3 chemokine contributes to specific CD8^+^ T cells migration, activation, and functionality. We used a neutralizing monoclonal antibody (anti-CXCR3) treatment approach. Our results show the increase in parasitemia and precocious mortality in anti-CXCR3-treated *T*. *cruzi*-infected mice. Our finding corroborate with the studies performed with *Toxoplasma gondii* and Herpes virus models, where, CXCR3-deficient mice die after infections with those pathogens [10,15].

The role of CXCR3 chemokine receptor guiding T lymphocytes to lymphoid tissues has been demonstrated in several models that included: allografts, autoimmune diseases, *T*. *gondii* infections, and the genital herpes virus [11,15,35]. In fact, we demonstrated that CXCR3 guides CD8^+^ T cells to the heart after infection with *T*. *cruzi*, since we observed a decreased in the frequency of those cells after anti-CXCR3 antibody treatment. In addition, the decrease numbers of CD8^+^ T cells lead to the increased parasite burden in the heart, which show the importance of CD8^+^ T cells in control *T*. *cruzi* infection.

Furthermore, we analyzed the number of specific CD8^+^ T cells in the spleen and we observed that CXCR3 mAb treatment decreased the number of specific CD8^+^ T cells in that tissue. However, the treatment did not alter significantly the absolute number of CD4^+^ and CD4^+^Foxp3^+^ T cells in the spleen, suggesting that the anti-CXCR3 treatment did not alter the migration of those cells. In spite of our result, the role of CXCR3 in CD4^+^ T cells migration has been shown in infection by *Plasmodium chabaudi* [36], however, in our model we believe that others chemokine might be involved in CD4^+^ T cells migration to spleen.

Regardless of the decrease in VNHRFTLV-specific CD8^+^ T cells in the spleen, we did not observe a decrease in specific CD8^+^ T cell proliferation or any changes in pro-apoptotic profile after the anti-CXCR3 treatment, indicating that the decrease in the number of specific CD8^+^ T cells was not due to the impaired proliferation or death pathways. Conversely, Thapa and colleagues have shown that CXCR3 is important to the T lymphocytes proliferation, however, CXCR3 was important to T cell proliferation in early days of infection by HSV-2 virus and here we evaluated the proliferation on day 15 after infection [37].

In addition, we observed a decrease in the number of IFN-γ producing CD8^+^ T cells and polyfunctionality in the anti-CXCR3 treated group. The lower polyfunctionality in anti-CXCR3 treated group was confirmed using OT-I infected mice with a transgenic parasite carrying OVA protein model. Once again, the anti-CXCR3 treatment decreased the number of polyfunctional SIINFEKL-CD8^+^ T cells. Corroborating with our results, T lymphocytes from CXCR3 deficient mice exhibited reduced IFN-γ production during viral and *Leishmania donovani* infection models [15,38].

Another important function triggered by CD8^+^ T cells is the directed cytotoxicity against infected cells [20,39,40]. That function was analyzed *in vivo* and anti-CXCR3 treated mice showed impairment in that function. However, we did not observe reduced production of granzyme B. Similarly, Hickman and colleagues, showed that specific CD8^+^ T cells from CXCR3-deficient mice had reduced cytotoxicity when compared to control group during the infection with vaccinia virus model. The authors suggested that that reduction in the cytotoxicity could be explained by the role of CXCR3 receptor in establish contact between CD8^+^ cytotoxic and infected antigen presenting cells [16].

We also evaluated the activation pattern of specific CD8^+^ T cells. After infection, those cells upregulated the expression of CD11a, CD44 and KLRG-1 and downregulated the expression of CD62L when compared to naïve CD8^+^ T cells [29]. However, specific CD8^+^ T cells from the anti-CXCR3 treated group downregulated the expression of CD11a, CD44, and KLRG-1 and upregulated CD62L molecule as well, when compared to specific CD8^+^ T cells from the isotype control group (Infected). Hu and collaborators demonstrated that mice infected with lymphocytic choriomeningitis virus (LCV), the colocalization of virus-specific CD8^+^ T cells with antigen in the spleen was dependent on CXCR3 expression [41], and we raised the hypothesis that the decreased in effector function of CD8^+^ T cells could be due to the lower prime by APCs. To address that, we evaluated which APCs were responsible for producing CXCL9 (MIG) and CXCL10 (IP-10) chemokines, the ligands for CXCR3. We observed that both plasmacytoid cells (pDCs) and Monocytes—Derived Dendritic cells (MO-DCs) produced CXCR3 ligands, however the pDCs expressed only CXCL10. The CXCL10 expression by MO-DCs and pDCs started very early and remained until day 20 after infection, which is consistent with the high expression of CXCR3 in specific CD8^+^ T cells, observed from day 5 to 30 after infection. We also observed a higher expression of CXCL10 in the heart of infected mice on day 15 after infection.

Furthermore, we observed that CXCL10-producing cells can interact with IFN-γ ^+^ cells after *T*. *cruzi* infection, and CXCL10^+^ produced by pDCs showed a higher interaction with CD8^+^ T cells than MO-DCs. Interestingly, the anti-CXCR3 treatment reduced the number of pDCs in the spleen and lymph node, and those cells also expressed CXCR3 receptor on the surface. Similarly, the reduction in the number of pDCs in the lymph node of CXCR3-deficient mice was observed during herpes virus type 2 infection [15]. In addition, expression of CXCR3 in pDCs was also demonstrated by other groups [42,43].

We believe that CXCR3 receptor is essential promote the encounter between pDCs and CD8^+^ T cells. The lower activation phenotyping and effector function of specific CD8^+^ T cells observed during anti-CXCR3 treatment can be explained by the lower quantity of pDCs in spleen and lymph node. Previously, our group demonstrated that during *T*. *cruzi* infection pDCs express co-stimulatory molecules and are responsible for activating anti-*T*. *cruzi* CD8^+^ T cells [44].

Overall, we have demonstrated that C57BL/6 mice died very quickly due to infection during the anti-CXCR3 treatment. Moreover, the number of specific CD8^+^ T-cells decreased as well as activation and polyfunctionality. We showed that pDCs and CD8^+^ T cells interacted *in vivo* and we believe that CXCR3 is important to pDCs and anti-*T*.*cruzi* CD8^+^ T cells encounter and subsequently, to antigen presentation by pDCs to CD8^+^ T cells. After priming, CD8^+^ T cells produce cytokines and kill the infected cells by cytotoxicity.

Altogether, our results indicate that CXCR3 plays a critical role in specific CD8^+^ T cells activation and migration. Understanding its role might help to promote the development of vaccines against intracellular parasites such as *Trypanosoma cruzi*.

## Supporting information

S1 Fig CD4 Foxp3^+^ T cells expressed CXCR3 after infection. a-Graph with the absolute number of CD4^+^ T cells in the spleen of naïve, isotype control and anti-CXCR3 groups. The number of total CD4^+^ T cells was calculated on day 15 after infection. b-Dot-plots represent the gate and the frequency of CD4^+^ Foxp3^+^ T cells in naïve, isotype control and anti-CXCR3 groups on day 15 after infection. c-Bar graph with the absolute number of CD4 Foxp3^+^ T cells. d-Histogram shows CD4 Foxp3^+^ T cells expressing CD25 molecule and bar graph with the MFI quantification of CD25 molecule expressed in CD4 Foxp3^+^ T cells, respectively. e-Histogram represents CD4 Foxp3^+^ T expressing CXCR3 molecule, and bar graph with MFI quantification of CXCR3 molecule on the surface of CD4 Foxp3^+^ T cells, respectively. Data are mean ± SD and are representative of 2 independent experiments with n = 3. The symbols indicate values that are statistically differences between the groups (ζP = 0.000661, ζζP < .01). The statistical analyses were carried out using One-way ANOVA, followed by Tukey post-hoc test).
(TIF)

S2 Fig SIINFEKL-specific CD8^+^ T cells treated with anti-CXCR3 decreased the polyfunctionality. OT-I mice were infected with 1x10^6^ forms of Y-OVA transgenic *T*. *cruzi* strain and treated with anti-CXCR3. On day 10 after infection, spleens were harvested and splenocytes were stimulated for 6 hours with SIINFEKL peptide. ICS staining was performed to quantify the cytokine production and degranulation by CD8^+^ T cells; we subdivided CD8 T cells that had performed 3, 2, or 1 function (s) at same time. a-Dot-plots graph show the frequency of specific CD8^+^ T cells from naïve, OT-I+Y-OVA+Isotype Control and OT-I+Y-OVA+anti-CXCR3 groups, double positive for: IFN-γ^+^ TNF-α^+^; CD107a^+^ and TNF-α^+^; IFN-γ^+^ and/or CD107a^+^IFN-γ^+^. b-The graph represents the percentage of specific CD8^+^ T cells that performed 3, 2, or 1 function. Boolean data were performed using FlowJo Software version 9.0. Data are mean ± SD and are representative of 2 independent experiments with n = 3.
(TIF)

S3 Fig CXCR3 antibody treatment did not alter the expression of some molecules on CD8^+^ T cells surface. The immunophenotyping of VNHRFTLV specific CD8^+^ T cells was performed in the spleen of naïve, Isotype control and anti-CXCR3 groups. We evaluated the expression of markers related to activation, homing and memory. a-The histogram graphs represent each molecule analyzed in specific CD8^+^ T cells in the spleen of naïve (grey line), Isotype Control (red line) and anti-CXCR3 (blue line) groups. Data are mean ± SD and are representative of 2 independent experiments with n = 3.
(TIF)
