# Correction: CXCR3 chemokine receptor contributes to specific CD8^+^ T cell activation by pDC during infection with intracellular pathogens

**DOI:** 10.1371/journal.pntd.0009326

**Published:** 2021-04-07

**Authors:** Camila Pontes Ferreira, Leonardo Moro Cariste, Isaú Henrique Noronha, Danielle Fernandes Durso, Joseli Lannes-Vieira, Karina Ramalho Bortoluci, Daniel Araki Ribeiro, Douglas Golenbock, Ricardo Tostes Gazzinelli, José Ronnie Carvalho de Vasconcelos

The second author’s name is spelled incorrectly. The correct name is: Leonardo Moro Cariste. The correct citation is: Pontes Ferreira C, Moro Cariste L, Henrique Noronha I, Fernandes Durso D, Lannes-Vieira J, Ramalho Bortoluci K, et al. (2020) CXCR3 chemokine receptor contributes to specific CD8^+^ T cell activation by pDC during infection with intracellular pathogens. PLoS Negl Trop Dis 14(6): e0008414. https://doi.org/10.1371/journal.pntd.0008414
