# Supplementary figures and images for: CXCR3 chemokine receptor contributes to specific CD8+ T cell activation by pDC during infection with intracellular pathogens
Source: PLoS Negl Trop Dis. 2020 Jun 23;14(6):e0008414. doi: 10.1371/journal.pntd.0008414 (PMC7337401; doi:10.1371/journal.pntd.0008414)

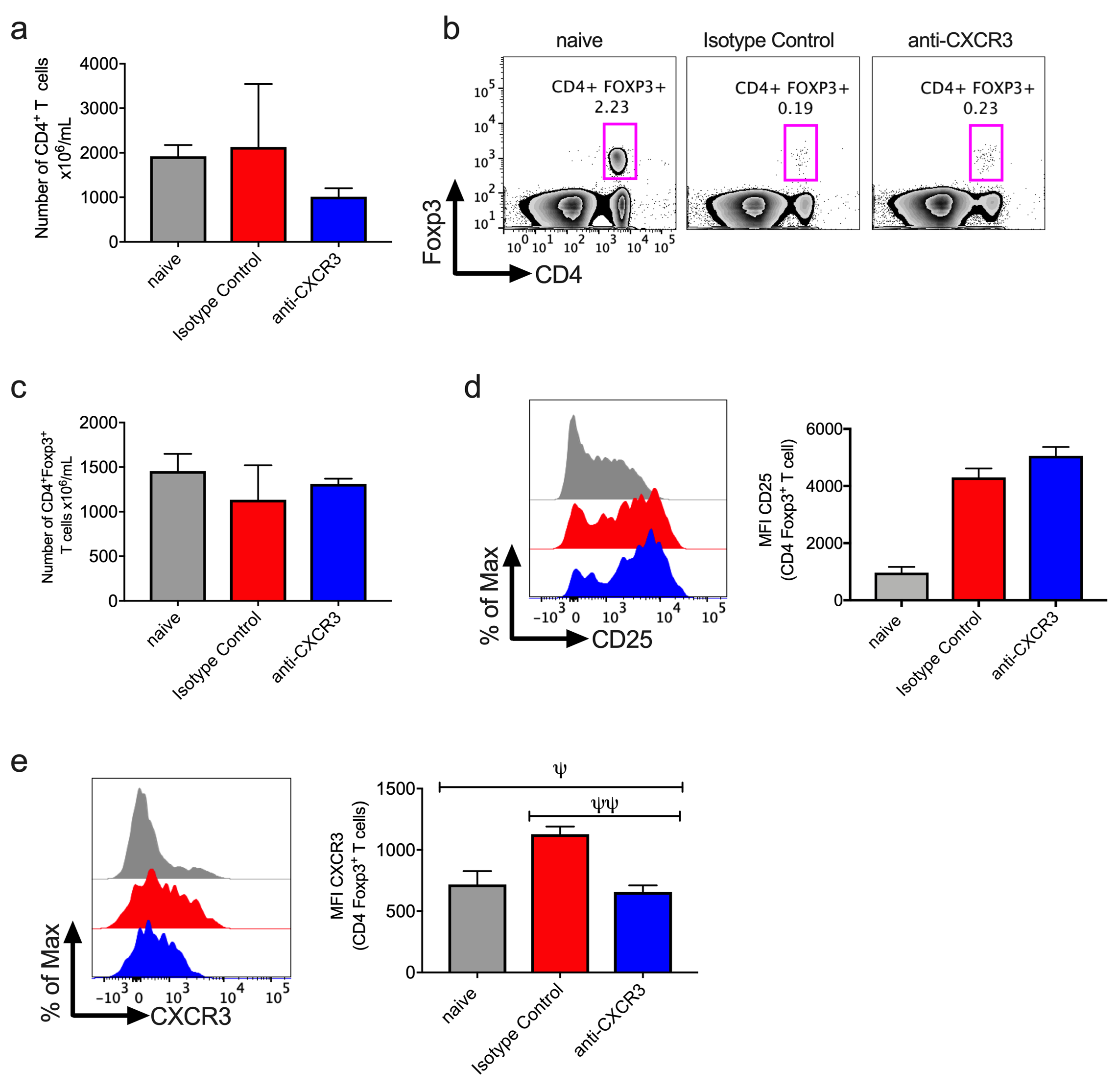

Supplement: S1 Fig — a-Graph with the absolute number of CD4+ T cells in the spleen of naïve, isotype control and anti-CXCR3 groups. The number of total CD4+ T cells was calculated on day 15 after infection. b-Dot-plots represent the gate and the frequency of CD4+ Foxp3+ T cells in naïve, isotype control and anti-CXCR3 groups on day 15 after infection. c-Bar graph with the absolute number of CD4 Foxp3+ T cells. d-Histogram shows CD4 Foxp3+ T cells expressing CD25 molecule and bar graph with the MFI quantification of CD25 molecule expressed in CD4 Foxp3+ T cells, respectively. e-Histogram represents CD4 Foxp3+ T expressing CXCR3 molecule, and bar graph with MFI quantification of CXCR3 molecule on the surface of CD4 Foxp3+ T cells, respectively. Data are mean ± SD and are representative of 2 independent experiments with n = 3. The symbols indicate values that are statistically differences between the groups (ζP = 0.000661, ζζP < .01). The statistical analyses were carried out using One-way ANOVA, followed by Tukey post-hoc test). (TIF) [file pntd.0008414.s001.tif]

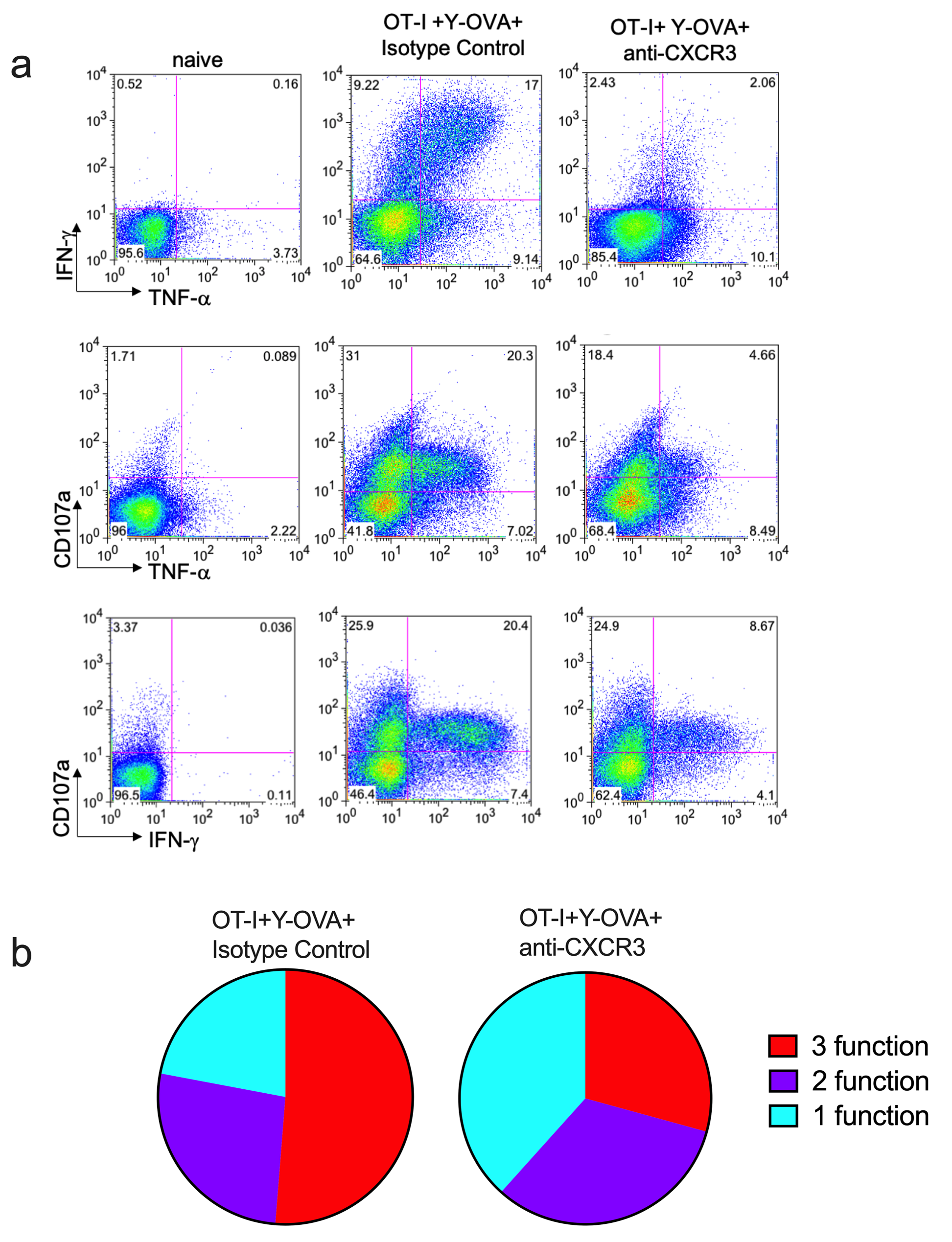

Supplement: S2 Fig — OT-I mice were infected with 1x10^6^ forms of Y-OVA transgenic T. cruzi strain and treated with anti-CXCR3. On day 10 after infection, spleens were harvested and splenocytes were stimulated for 6 hours with SIINFEKL peptide. ICS staining was performed to quantify the cytokine production and degranulation by CD8+ T cells; we subdivided CD8 T cells that had performed 3, 2, or 1 function (s) at same time. a-Dot-plots graph show the frequency of specific CD8+ T cells from naïve, OT-I+Y-OVA+Isotype Control and OT-I+Y-OVA+anti-CXCR3 groups, double positive for: IFN-γ+ TNF-α+; CD107a+ and TNF-α+; IFN-γ+ and/or CD107a+IFN-γ+. b-The graph represents the percentage of specific CD8+ T cells that performed 3, 2, or 1 function. Boolean data were performed using FlowJo Software version 9.0. Data are mean ± SD and are representative of 2 independent experiments with n = 3. (TIF) [file pntd.0008414.s002.tif]

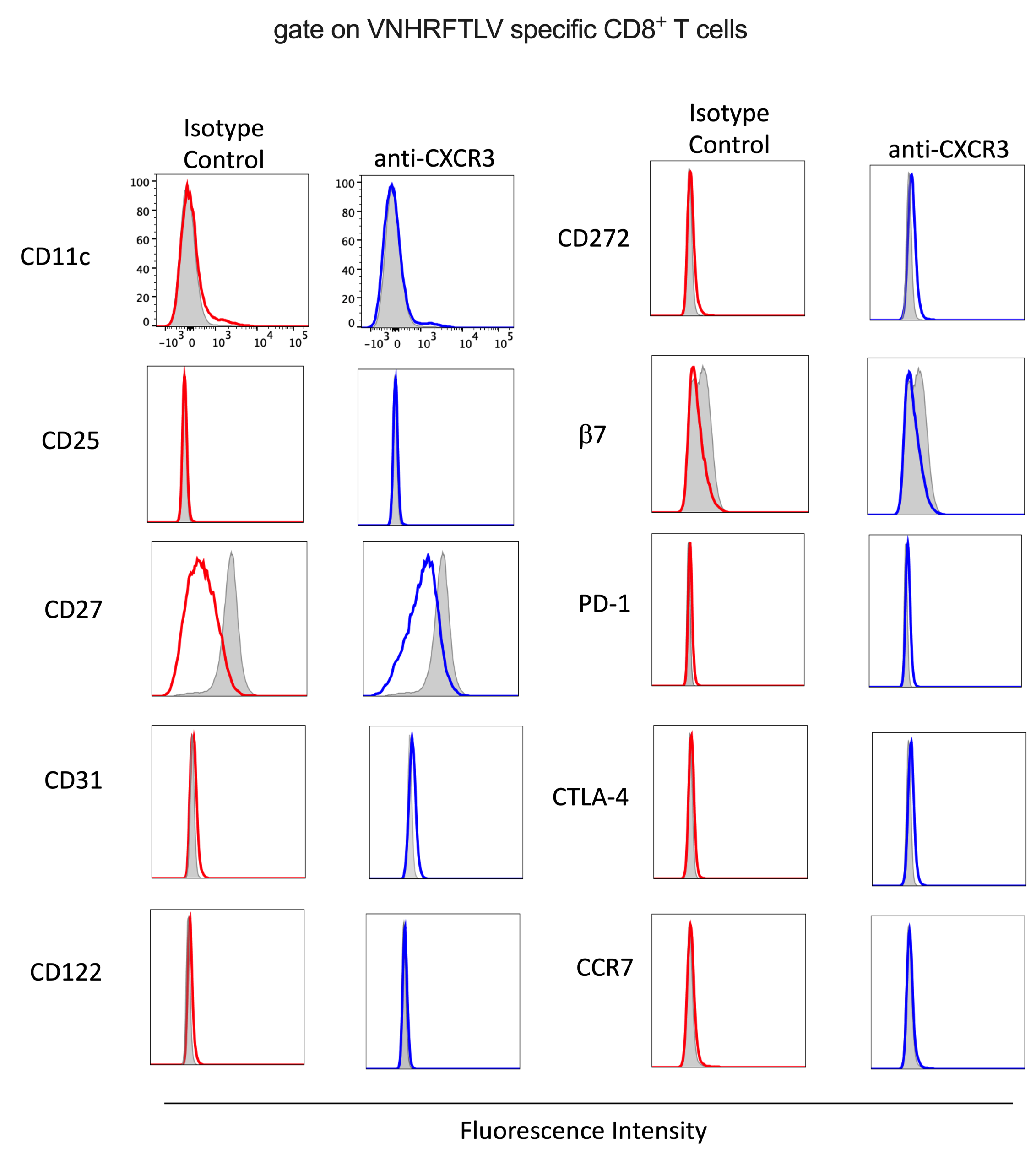

Supplement: S3 Fig — The immunophenotyping of VNHRFTLV specific CD8+ T cells was performed in the spleen of naïve, Isotype control and anti-CXCR3 groups. We evaluated the expression of markers related to activation, homing and memory. a-The histogram graphs represent each molecule analyzed in specific CD8+ T cells in the spleen of naïve (grey line), Isotype Control (red line) and anti-CXCR3 (blue line) groups. Data are mean ± SD and are representative of 2 independent experiments with n = 3. (TIF) [file pntd.0008414.s003.tif]
